# RNA interference is essential to modulating the pathogenesis of mosquito-borne viruses in the yellow fever mosquito *Aedes aegypti*

**DOI:** 10.1073/pnas.2213701120

**Published:** 2023-03-09

**Authors:** Glady Hazitha Samuel, Tyler Pohlenz, Yuemei Dong, Nese Coskun, Zach N. Adelman, George Dimopoulos, Kevin M. Myles

**Affiliations:** ^a^Department of Entomology, Minnie Belle Heep Center, Texas A & M University, College Station, TX 77843-2475; ^b^W. Harry Feinstone Department of Molecular Microbiology and Immunology, Bloomberg School of Public Health, Johns Hopkins University, Baltimore, MD 21205-2179

**Keywords:** mosquito, RNAi, Dicer, virus, immunity

## Abstract

While it has long been known that the transmission of mosquito-borne viruses depends on the establishment of persistent and nonlethal infections in the invertebrate host, specific roles for the insects’ antiviral immune pathways in modulating the pathogenesis of viral infections is the subject of speculation and debate. Here, we show that a loss-of-function mutation in the *Aedes aegypti* *Dicer-2* (*Dcr-2*) gene renders the insect acutely susceptible to a disease phenotype upon infection with pathogens in multiple virus families associated with important human diseases. Additional interrogation of the disease phenotype demonstrated that the virus-induced pathology is controlled through a canonical RNA interference (RNAi) pathway, which functions as a resistance mechanism. These results suggest comparatively modest contributions of proposed tolerance mechanisms to the fitness of *A. aegypti* infected with these pathogens. Similarly, the production of virus-derived piwi-interacting RNAs (vpiRNAs) was not sufficient to prevent the pathology associated with viral infections in *Dcr-2* null mutants, also suggesting a less critical, or potentially secondary, role for vpiRNAs in antiviral immunity. These findings have important implications for understanding the ecological and evolutionary interactions occurring between *A. aegypti* and the pathogens they transmit to human and animal hosts.

## Significance

Models of mosquito-borne disease transmission illustrate that the longevity of the insect vector is a particularly powerful factor in the calculation of basic reproduction number (R_0_). The mosquito must survive long enough after ingesting the pathogen to deliver infectious bites. Significant levels of pathogen-associated mortality in this host would have a detrimental impact on transmission. Here, we show that *Aedes aegypti* with a genetic lesion in Dicer-2 are acutely susceptible to multiple viruses associated with human disease, but not other types of infections. Further, we demonstrate that the pathology associated with these infections is primarily controlled through an RNA interference pathway that functions as a resistance mechanism. These results reveal insights into the transmission of many important diseases.

Calculating the potential spread of mosquito-borne diseases differs from that of other communicable diseases because of the involvement of an insect vector, which can have a large impact on the number of additional persons that may become infected from an already infected host. Even the earliest models of mosquito-borne disease transmission made clear that the longevity of the vector host is a particularly powerful factor in the calculation of the basic reproduction number or R naught (R_0_) (1). While there are multiple reasons for this, an important one is that after the mosquito acquires a pathogen it must survive long enough to deliver infectious bites. Any significant reduction in the lifespan of the insect, through disease and mortality, would reduce the time available for pathogen development during an extrinsic incubation period and limit fecundity, ultimately decreasing vectorial capacity. In the 1970s, the existence of a phenomenon restricting virus replication was postulated in order to explain the resistance of mosquito cells to any apparent cytopathology after infection with arthropod-borne viruses (arboviruses) (2, 3). At the time, it was suggested that this resistance might be tenuous, representing a weak point in the transmission cycle that could potentially be exploited for disease control (2). However, the idea remained theoretical until the discovery of RNA interference (RNAi) pathways in nearly all eukaryotic organisms.

In plant and invertebrate organisms, Dicer proteins have been shown to function as distinct pattern recognition receptors that process double-stranded RNA (dsRNA), a pathogen-associated molecular pattern commonly associated with viral infection (4–7). *Drosophila melanogaster* with genetic lesions in Dicer-2 (*Dcr-2*) were essential to demonstrating the nuclease’s role in the production of virus-derived siRNAs and the initiation of a protective antiviral immune response (6, 8, 9). Exogenous sources of dsRNA are processed into short interfering RNA (siRNA) duplexes [~21 nucleotides in length] by the RNase III enzyme Dcr-2 (6, 8, 10). These siRNAs are then incorporated into an RNA-induced silencing complex (RISC) (11). One strand of the siRNA duplex acts as a guide, providing sequence specificity to the RISC, while the other “passenger” strand is removed from the activated RISC (12, 13). An essential component of the RISC, Argonaute-2 (AGO-2), possesses “slicer” activity (14). The guide strand directs AGO-2 and the RISC to complementary RNAs in the cell, leading to their specific cleavage and degradation. An antiviral role for the siRNA pathway of the prolific disease vector, *Aedes aegypti,* is also well supported; reviewed in refs. 15 and 16. Knocking down components of the RNAi pathway in mosquito cell lines and adults has been correlated with elevated levels of virus replication (15, 16). Studies suppressing or genetically ablating virus-derived siRNAs in cell lines and adults have confirmed the molecular and genetic basis for the increases in replication (15–17). However, studies in *A. aegypti* have also revealed a number of differences with RNAi pathways present in *Drosophila* and other organisms.

There is evidence to suggest that a ping-pong-dependent piwi-interacting RNA (piRNA) pathway, which functions independently of Dcr-2, may also target RNA viruses in the soma of disease vector mosquito species (18–23)*.* A characteristic enrichment of adenine residues at the 10th position, or so called A10 nucleotide (nt) bias, in ping-pong dependent piRNAs has long been the hallmark of products derived by this mechanism in the *Drosophila* germline (24). Another characteristic of mature piRNAs is the tendency to begin with a uridine (1U), which may result from either “phasing” or the fact that some piRNAs serve as guide sequences, generating additional piRNAs from homologous strands (24). As cleavages mediated by guide piRNAs occur between their 10th and 11th nucleotides, guide sequences possessing an A10 bias will ultimately give rise to processed target piRNAs with the 1U tendency (24). In *Drosophila,* this process has been shown to generate a signature 10 nucleotide overlap in piRNAs derived from opposite messenger RNA (mRNA) strands, which may be detected in large sequencing datasets through bioinformatic approaches (25–27).

Specific examples of pathogens transmitted by the mosquito *A. aegypti* include Zika virus, dengue virus serotypes 1 to 4 (DENV 1-4), Japanese encephalitis virus, yellow fever virus (YFV), and chikungunya virus (CHIKV), which are collectively responsible for hundreds of millions of cases of human morbidity each year. Although the transmission of these viruses depends on the establishment of a persistent, nonlethal infection in the insect vector host, the mechanism or mechanisms by which pathogenesis is modulated in *A. aegypti* remains a topic of speculation and debate. Inhibiting the production of siRNAs with the well-known flock house virus suppressor of RNA silencing, B2, was shown to increase mortality in mosquitoes infected with a recombinant Sindbis virus (SINV), but these experiments could not entirely rule out the possibility that the observed phenotype in mosquitoes might be due to some hitherto undescribed function of the B2 protein (28). Further, it has been proposed that virus-derived DNA (vDNA) may play a role in the establishment of persistent arboviral infections by affecting the tolerance of mosquitoes to these pathogens (29, 30). Inhibiting endogenous reverse transcriptases in disease vector mosquitoes infected with alphavirus and flavivirus pathogens was shown to decrease production of viral siRNAs and piRNAs, and increase mortality (29). No doubt the absence of concomitant increases in the replication of these viruses led the authors to hypothesize the presence of vDNAs affected tolerance rather than resistance to the pathogens tested (29). Thus, a number of questions persist regarding the apparent absence of overt disease and mortality in mosquitoes infected with arboviruses, the etiological agents of many important human diseases.

Here, we interrogated the role of *A. aegypti* Dcr-2 in the resistance of the insect host to pathogens associated with human disease. This was done by measuring viral loads in *Dcr-2* null mutant *A. aegypti*, along with the secondary phenotype of survival, which was taken as representing the effects of the viral infection on the fitness of the vector host. Similar approaches have been successfully employed in the identification of host factors (e.g., Toll pathway and antimicrobial peptides) contributing to the resistance of *D. melanogaster* to various fruit fly pathogens (6, 8, 9, 31–35). We show that *Dcr-2* null *A. aegypti* are acutely susceptible to a disease phenotype upon infection with multiple arbovirus pathogens, and demonstrate that the pathology associated with these infections is primarily controlled in the insect through the canonical siRNA pathway, which functions as a resistance mechanism. Our results suggest that the contributions of proposed tolerance mechanisms to the fitness of *A. aegypti* infected with these pathogens are comparatively modest. Similarly, the production of virus-derived piwi-interacting RNAs (vpiRNAs) was not sufficient to prevent a disease phenotype in the arbovirus-infected *Dcr-2* null mosquitoes, also suggesting a less critical, or potentially secondary, role for vpiRNAs in antiviral immunity. These findings have important implications for understanding the transmission of pathogens that impose a significant burden on global public health.

## Materials and Methods

### Mosquitoes and Infections.

The generation of *Dcr-2* null mutant *A. aegypti* by gene editing has been described (36). The generation of the transgenic *A. aegypti* “sensor” strain has been described (37). *Dcr-2* mutants were screened for expression of eye-specific enhanced green fluorescent protein (EGFP) 6 d after emergence of adults. All mosquito infections were performed by injection of 0.5 µL (10^6^ pfu/mL) YFV (ASIBI), DENV-2, DENV-4, or SINV prepared in Dulbecco’s modification of Eagle’s medium (DMEM) supplemented with 10% fetal bovine serum, nonessential amino acids, L-glutamine, and antibiotics into the thoraces of adult mosquitoes. Mock-infected mosquitoes were inoculated with the same volume of DMEM. Liverpool strain or wild-type sibling mosquitoes (scored as negative for EGFP expression) served as controls. Three-to-five-day-old *A. aegypti* were fed on a blood meal containing 6.7 × 10^6^ pfu/mL SINV. Blood meals were prepared by mixing equal volumes of defibrinated sheep erythrocytes and virus suspension. Blood meals were heated to 37 °C and administered to mosquitoes with a Hemotek feeding apparatus. Mosquitoes were permitted to feed for 1 h and then fully engorged females were separated and held in individual chambers. A portion of each infectious blood meal was frozen and back titers determined by plaque assay. Liverpool, *Dcr-2* null mutant, or wild-type sibling *A. aegypti* strains were incubated at 28 °C and 80% relative humidity, with a 14/10-h day/night light cycle. Assays for infectivity were performed as described previously (38). For systemic bacterial challenge assays, laboratory standard strains of *E. coli* (DH5a) and *Staphylococcus aureus* (*S. aureus*), representing gram-negative and gram-positive bacteria, respectively, were used as previously described (39, 40). Briefly, bacteria were cultured in Luria–Bertani medium at 37 °C at 250 rpm overnight for 16 h. This was followed by two washes with phosphate buffered saline (PBS) before resuspending cell pellets in PBS at an OD600 = 2.0. Infections were performed by injection (Nanoject apparatus; Drummond Scientific) of 69 nL bacterial suspension into the thoraces of adult mosquitoes. Mock-infected mosquitoes were inoculated with the same volume of PBS buffer as a negative control.

### RNA Isolation and Detection.

RNA was isolated from pools (n = 5) of adult female mosquitoes 96 h after infection with Tri Reagent RT (Molecular Research Center) according to the manufacturer’s instructions. The levels of positive sense viral RNAs present in the samples were determined by strand-specific quantitative real-time PCR (RT-qPCR) using Taqman assays (Life Technologies) and a standard curve, as described previously (41). The specific primers and probes used were as follows: SINV F, 5′-ATCACAATTGGCAACGAGAAGAG-3′, SINV R, 5′-CTGTGGGTTCGGAGAATAGTGG-3′, SINV probe, 5′-CTAAAAGCAGCCGAACTC-3′; YFV F, 5′-GGTTCCATGAGCGTGGCTAT-3′, YFV R, 5′-GCGCAGCAGCGTAGTAACAC-3′, YFV Probe, 5′-CAAGCTGGAAGGTAGGGTGAT-3′; DENV-2 F, 5′-CCAGTGGAATCATGGGAGGA-3′, DENV-2 R, 5′-CCCTGCTTGTTAGCCCAATC-3′, DENV-2 probe, 5′-TGAGCCGCACCATTGGTCTTCTCT-3′; DENV-4 F, 5′-CCCATCACTA​ACAAAACGCAG-3′, DENV-4 R, 5′-TCTAACCTCTAGTCCTTCCACC-3′, DENV-4 probe, 5′-TACAGCTTCCTCCTGGCTTCGG-3′*.*

### Small RNA Preparation and Analysis.

Libraries were prepared from the same total RNA isolated for RT-qPCR. RNAs 18 to 40 nt in length were isolated by polyacrylamide gel electrophoresis separation and prepared for sequencing with the Truseq small RNA sample prep kit (Illumina) according to the manufacturer's instructions. All independent biological replicates in a cohort were multiplexed and sequenced on a single lane of a HiSeq (Illumina) flow cell by the Next Generation Sequencing and Microarray Core Facility, The Scripps Research Institute, La Jolla, California. Bioinformatic analysis of libraries was performed as described previously (19). Briefly, 3′ adapter sequences were trimmed with Fastx_clipper (http://hannonlab.cshl.edu/fastx_toolkit/) and identifiable noncoding RNA sequences (rRNAs, tRNAs, snRNAs, snoRNAs, etc.) removed with Bowtie (42). Remaining reads were then mapped to the appropriate reference genome using the same program (v-mode, permitting 1 mismatch). The edgeR Bioconductor software package (https://bioconductor.org/packages/release/bioc/html/edgeR.html) was used to determine differential expression between replicate datasets. Raw sequence counts with less than ten copies per million reads were excluded from the input, with all identifiable reads (vsRNAs, miRNAs, TE-derived small RNAs, endo-siRNAs, etc.) being used to normalize the datasets through the TMM method (43). Nucleotide biases were analyzed with R. Small RNAs mapping to YFV were analyzed for a 10-nt overlap with a previously published algorithm (27). As the typical overlap between a pair of sense and antisense piRNAs occurs over the first 10 nt of the 5′-end of each sequenced read, a range of no more than 20 was specified. For the presentation of figures, all datasets being directly compared were normalized to the library containing the smallest number of identifiable reads. This was done by randomly selecting the same number of small RNA reads from each of the larger datasets, creating a minimum of three datasets of equal size for further analysis. Representative replicates were then selected for figure presentation.

### Statistical Analysis.

RT-qPCR results were analyzed with a two-tailed unpaired *t* test, minimum three independent biological replicates and three technical machine replicates. Infection data were analyzed with a two-tailed unpaired *t* test, minimum three independent biological replicates. Survival curves were generated by the Kaplan–Meier method, with data for either two or three groups being compared by the log-rank (Mantel–Cox) test. Differential expression between normalized replicate small RNA datasets was analyzed with the edgeR software package as described above. Z-scores for the number of small RNA pairs found with overlaps ranging from 1-20 nucleotides were calculated with a previously published formula (27).

## Results

### A Genetic Lesion Introduced into *A. aegypti Dcr-2* Is a Loss-of-Function Mutation.

**Fig. 1.**
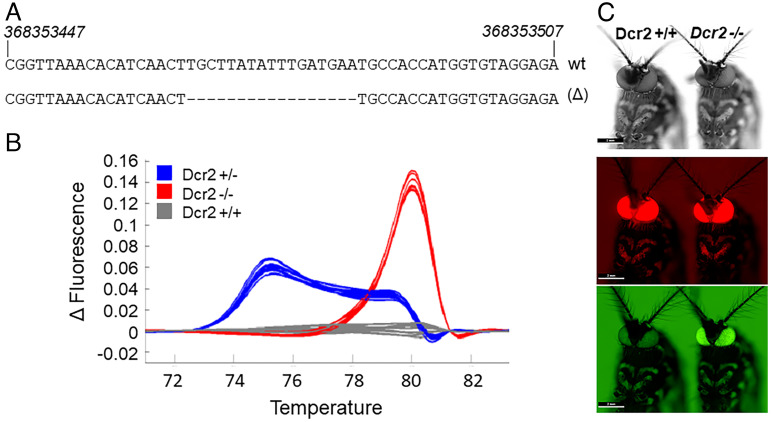
A genetic lesion introduced into *A. aegypti Dcr-2* is a loss-of-function mutation. (*A*) A 17 base pair deletion in exon 5 of *Dcr-2* introduced through gene editing. (*B*) Mosquitoes with the 17 base pair germ line deletion identified by high-resolution melt analysis (HRMA). Representative melting curves of sibling mosquitoes with heterozygous (*Dcr-2*^*+/−*^), homozygous (*Dcr-2*^*−/−*^) or wild-type (*Dcr-2*^*+/+*^) genotypes at the location of the 17 base pair deletion are shown in blue, red, and gray, respectively. (*C*) Sibling transgenic sensor mosquitoes with either wild-type or homozygous mutant *Dcr-2* alleles. The representative images show expression of DsRed indicating that both sibling *A. aegypti* contain the transgenic sensor construct. Mosquitoes with the wild-type *Dcr-2* alleles (shown on the *Left*) effectively silence EGFP expression in the transgenic sensor line. However, mosquitoes homozygous for the mutant alleles (shown on the *Right*) are unable to efficiently process the EGFP-specific dsRNA substrate formed by the inverted repeat sequence expressed from the sensor construct and effective silencing of EGFP is prevented, resulting in the strong EGFP+ phenotype shown.

The *A. aegypti* *Dcr-2* (AAEL006794) gene was edited with a pair transcription activator-like effector nucleases generating a 17 base pair deletion in exon 5 (Fig. 1*A*). Mosquitoes with this germ-line mutation, identified through high-resolution melt analysis (HRMA; Fig. 1*B*), were subsequently outcrossed to wild-type Liverpool strain mosquitoes over six successive generations. To confirm that the 17 base pair deletion was in fact a loss-of-function mutation, additional outcrosses were performed with a previously described transgenic *A. aegypti* sensor strain (37). Briefly, this *kmo* mutant (white eye) strain is transformed with a construct containing three independent, but identical, eye-specific 3xP3 promoters. The first promoter expresses the red fluorescent protein derived from *Discosoma sp.* (DsRed), which serves as a marker of transformation. The second 3xP3 promoter expresses EGFP. While the expression of EGFP from this promoter would normally result in an eye-specific EGFP**+** phenotype in this white-eyed strain, the third 3xP3 promoter in the series expresses an inverted repeat sequence with homology to a portion of the EGFP mRNA. Thus, the eye-specific EGFP**+** phenotype is only observed upon inactivation or impairment of RNA silencing, which prevents the siRNA pathway from processing the dsRNA substrate formed by the inverted repeat sequence and silencing EGFP (37). Strong eye-specific expression of EGFP was observed in sensor strain mosquitoes homozygous for the edited *Dcr-2* allele (Fig. 1*C*), indicating that the 17 base pair deletion is in fact a loss-of-function mutation. Interestingly, heterozygous mutants were also able to silence EGFP, indicating that the *AaDcr-2* gene is haplosufficient, with a single functional allele being dominant (*SI Appendix*, Fig. S1). Thus, eye-specific expression of EGFP also proved to be useful as a phenotypic marker for screening and selecting adult mosquitoes homozygous for the mutant *Dcr-2* allele. The survival of homozygous null mutants, identified through eye-specific EGFP expression levels, over a 2-wk time period was not significantly different from that of wild-type siblings or Liverpool strain *A. aegypti*, indicating that the loss of function mutation had little or no adverse effect on the vigor of the mutants in the absence of microbial challenge (*SI Appendix*, Fig. S2). Further, challenge of *Dcr-2* null mutant *A. aegypti* with both gram-negative and gram-positive bacteria did not reveal a general susceptibility to nonviral pathogens (*SI Appendix*, Fig. S3). However, consistent with results reported in a previous study with *D. melanogaster* (44), *Dcr-2* null mutants demonstrated increased susceptibility to the gram-positive bacteria, *S. aureus* (*SI Appendix*, Fig. S3). Thus, additional studies may be merited in order to investigate possible interactions between Dcr-2 and the Toll pathway in *A. aegypti*.

### The siRNA Pathway Modulates the Pathogenesis of SINV in *A. aegypti*.

**Fig. 2.**
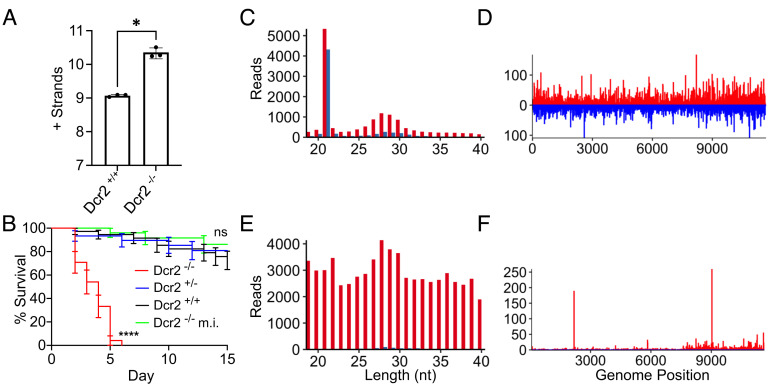
The siRNA pathway modulates the pathogenesis of SINV in *A. aegypti*. (*A*) RT-qPCR results showing the accumulation of positive strand viral RNA (+ strands) as an indication of viral load in wild-type or *Dcr-2* null sibling mosquitoes 4 d after infection with SINV (~500 pfu injected into individual mosquitoes). The *y* axis is labeled with log10 values. Error bars indicate the SEM calculated from three independent biological replicates (n = 5). Significance was determined by a two-tailed *t* test performed on base values, with * indicating a *P* value < 0.05. (*B*) Survival of sibling mosquitoes with wild-type (black line), heterozygous mutant (blue line), or homozygous mutant (red line) *Dcr-2* alleles after infection with SINV. The survival of homozygous *Dcr-2* null mutants that were mock infected is also shown (green line). Survival curves represent cohorts of ≥ 30 adult female mosquitoes individually injected with ~500 pfu of virus. Significance was determined by a Mantel–Cox log-rank test, with ns representing not significant and **** indicating a *P* value < 0.0001. (*C*) Distribution by length of small RNAs mapping to the infecting SINV genome in wild-type siblings. Results are representative of three independent biological replicates. Small RNAs were sequenced from the same pools of mosquitoes used to quantify viral load. Red bars represent reads derived from virus positive strands, while blue bars represent sequences derived from negative strands. (*D*) Density plots demonstrating the distribution of loci in the SINV genome generating 21-nt reads in wild-type siblings. (*E*) Distribution by length of small RNAs mapping to the SINV genome in Dcr-2 null siblings. (*F*) Density plots demonstrating the distribution of loci in SINV generating 21-nt reads in *Dcr-2* null siblings.

In order to evaluate both the role of the siRNA pathway and piRNA pathway, in protecting adult mosquitoes from the pathogenic effects of arbovirus infections, we infected *Dcr-2* null and wild-type sibling *A. aegypti* with SINV. In comparison to wild-type siblings, *Dcr-2* null mutants exhibit a clear disease phenotype (Movie S1), characterized by significantly elevated levels of virus replication (Fig. 2*A*) and mortality (Fig. 2*B*). Notably, the mortality of wild-type siblings infected with SINV is statistically indistinguishable from that of heterozygous mutants infected with equivalent titers of the same virus (Fig. 2*B*), indicating that a single functional *Dcr-2* allele is sufficient to prevent the disease phenotype. Importantly, the survival of mock-infected *Dcr-2* null mutants is not significantly different from that of heterozygous mutant or wild-type sibling mosquitoes infected with SINV (Fig. 2*B*), indicating that the mortality observed in SINV-infected null mutants is due to the virus infection rather than the inoculation itself.

We next sequenced and analyzed small RNAs present in *Dcr-2* null and wild-type siblings infected with SINV. Consistent with previously published descriptions of alphavirus-derived small RNAs in Aedes species and cell lines (19–22), two distinct populations of viral small RNAs were observed in the wild-type sibling mosquitoes (Fig. 2*C*). The first being characterized by a distinct peak at 21 nucleotides, generated from both the positive and negative strands of the virus in approximately equal proportions, indicating these to be the biogenic products of the siRNA-based antiviral response (Fig. 2 *C* and *D*). The second population of viral small RNAs in these mosquitoes was characterized by a smaller, but broader based peak, centered around 27 to 29 nucleotides (Fig. 2*C*). These small RNAs exhibited a prominent positive-strand bias, suggesting they are the products of the piRNA pathway. Bioinformatic analysis of SINV-derived small RNAs ranging from 23 to 30 nucleotides in length confirmed these to be products of the piRNA pathway, exhibiting the signature 10 nucleotide overlap, A10 bias, and enrichment for 1U on the opposite anti-sense strand (*SI Appendix*, Fig. S4).

Previous work in *Dcr-2* null mutant *A. albopictus* cell lines suggested that an antiviral response directed solely by vpiRNAs might be capable of modulating alphavirus pathogenesis in the absence of siRNA production (19). While we did observe increased production of virus-derived products that were 23 to 30-nt in length in *Dcr-2* null *A. aegypti* infected with SINV (Fig. 2*E*), which we confirmed to be vpiRNAs (*SI Appendix*, Fig. S4)*,* this increase was not sufficient to prevent mortality, at least not to the same extent observed in wild-type or heterozygous siblings infected with equivalent titers of virus (Fig. 2*B*). However, we are unable to exclude the possibility that additional mortality might occur, particularly at earlier time points, in the absence of the increased vpiRNA production that was observed. An exceedingly small number of 21-nt small RNAs produced in the absence of a functional siRNA pathway were mapped to specific SINV loci from whence they were generated (Fig. 2*F*). These 21-nt products, produced independently of Dcr-2, exhibited asymmetry and clustering consistent with previous descriptions of 21-nt vpiRNAs in *Dcr-2* null cell lines infected with CHIKV (19). However, bioinformatic analysis for specific nucleotide biases or overlapping sequences failed to identify prominent hallmarks of piRNA biogenesis, other than a relatively modest enrichment for uridine and adenine at the first nucleotide position of anti-sense 21-nt small RNAs derived from SINV in the *Dcr-2* null mosquitoes (*SI Appendix*, Fig. S4). While these products cannot be confirmed as piRNAs, the extremely limited number of reads may have prevented any meaningful analysis of overlapping pairs, and also may have prevented the identification of specific biases as well.

**Fig. 3.**
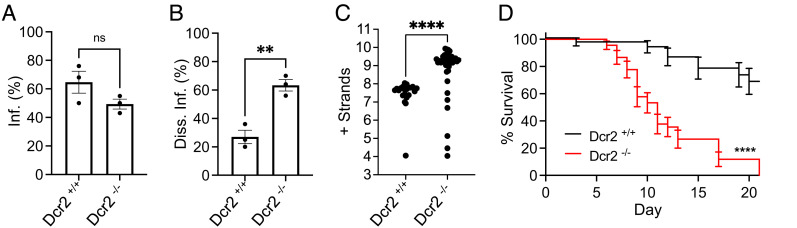
The *A. aegypti* siRNA pathway limits virus dissemination from the midgut and modulates pathogenesis in mosquitoes that develop systemic infections. (*A*) Prevalence of midgut infections in *Dcr-2* null or wild-type sibling mosquitoes ingesting a blood meal containing 6.7 × 10^6^ pfu/mL of SINV, assayed 6 d after feeding. Error bars indicate the SEM calculated from three independent biological replicates (n ≥ 20). Differences in the prevalence of infections between the two different groups of replicate cohorts were determined by a two-tailed *t* test, with ns representing not significant. (*B*) Prevalence of *Dcr-2* null or wild-type sibling mosquitoes with disseminated or systemic SINV infections, assayed 6 d after feeding. Error bars indicate the SEM calculated from three independent biological replicates (n ≥ 10). Differences in the prevalence of infections between the two different groups of replicate cohorts were determined by a two-tailed *t* test, with ** representing a *P* value < 0.01. (*C*) RT-qPCR results quantifying positive strand viral RNA (+ strands), i.e., viral load, in wild-type or *Dcr-2* null sibling mosquitoes with disseminated SINV infections. Scatter plots of base values obtained from individual mosquito bodies (n ≥ 22). The *y* axis is labeled with log10 values. Significance was determined by a two-tailed *t* test performed on base values, with **** indicating a *P* value < 0.0001. (*D*) Survival of sibling mosquitoes with wild-type (black line) or homozygous mutant (red line) *Dcr-2* alleles and systemic SINV infections. Survival curves represent cohorts of ≥ 36 adult female mosquitoes following per os challenge with 6.7 × 10^6^ pfu/mL of SINV. Significance was determined by a Mantel-Cox log-rank test, with **** representing a *P* value < 0.0001.

In order to confirm that the disease phenotype observed previously was not somehow specific to the route of infection, we next administered SINV to *Dcr-2* null and wild-type sibling mosquitoes in an infectious blood meal. Loss of functional Dcr-2 did not increase the prevalence of SINV infections in comparison with wild-type siblings 6 d after virus ingestion (Fig. 3*A*). However, the prevalence of disseminated or systemic virus infections in the *Dcr-2* null cohort was significantly higher than observed in the wild-type sibling mosquitoes at the same time point (Fig. 3*B*). This suggests that the siRNA pathway is an important determinant of whether or not the virus successfully escapes the midgut to establish a systemic infection, which is essential for disease transmission. These results are consistent with those of a previous study in which knocking down the expression of AGO-2 in *A. aegypti* did not increase the prevalence of midgut infections with DENV (3 to 4 d post infection), but did result in increased dissemination of the virus at 8 d post infection (45). Importantly, *Dcr-2* null mutants with disseminated SINV infections also exhibited a clear disease phenotype, which was again characterized by significantly elevated levels of virus replication and mortality in comparison to wild-type sibling mosquitoes (Fig. 3 *C* and *D*). The survival of the remaining *Dcr-2* null mutant mosquitoes ingesting the infectious blood meal (i.e., excluding *Dcr-2* null mutants with systemic infections) was statistically indistinguishable from that of wild-type siblings ingesting an equivalent titer of SINV (*SI Appendix*, Fig. S5). This result indicates that pathogenesis requires escape from the midgut and the establishment of a systemic infection. Thus, the siRNA pathway appears to be essential both for limiting dissemination of the virus from the midgut tissue, and modulating the pathogenesis of the systemic mosquito infections necessary for disease transmission. Interestingly, the pathogen load in *Dcr-2* null mutants that succumbed to systemic infections 6 to 10 d after ingesting the virus did not significantly differ from those of mutants succumbing 11 to 21 d after virus ingestion (*SI Appendix*, Fig. S6). Thus, the observed differences in the longevity of the systemically infected mutants suffering under similar levels of viral burden may represent evidence of genetic variation in tolerance mechanisms, and in the future, this would be interesting to investigate further.

### The siRNA Pathway Modulates the Pathogenesis of Flavivirus Infections in *A. aegypti*.

**Fig. 4.**
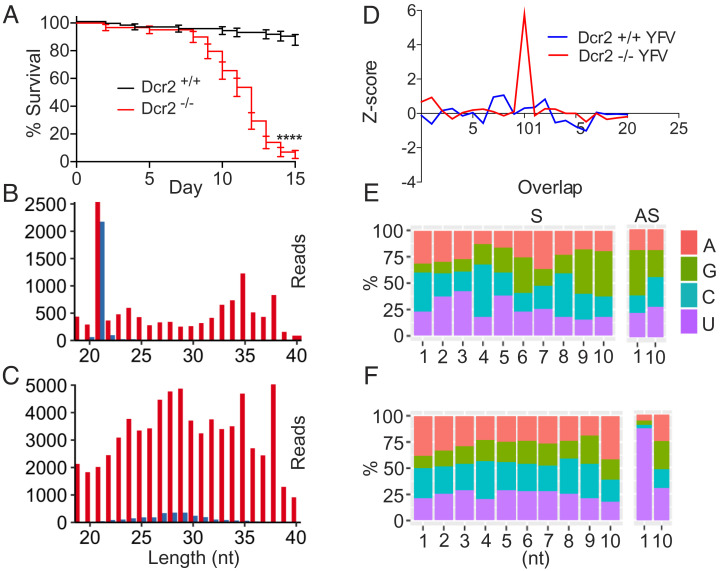
The siRNA pathway modulates the pathogenesis of YFV infections in *A. aegypti*. (*A*) Survival of wild-type (black line) or *Dcr-2* null (red line) sibling mosquitoes infected with YFV. Survival curves represent cohorts of ≥ 30 adult female mosquitoes injected with ~500 pfu of virus. Significance was determined by a Mantel-Cox log-rank test, with **** indicating a *P* value < 0.0001. (*B*) Distribution by length of small RNAs mapping to the infecting YFV genome in wild-type siblings. Results are representative of three independent biological replicates. Red bars represent reads derived from virus positive strands, while blue bars represent sequences derived from negative strands. (*C*) Distribution by length of small RNAs mapping to YFV in *Dcr-2* null siblings. (*D*) Z-scores for the number of small RNA pairs with the length of nucleotide overlap indicated, determined from all 23 to 30-nt reads mapping to YFV in either wild-type (shown in blue) or *Dcr-2* null (shown in red) sibling mosquitoes. (*E*) Stacked bar graph showing individual nucleotide biases present at the positions indicated in all 23 to 30-nt sequences derived from sense (S) or anti-sense (AS) strands of YFV in wild-type siblings*.* (*F*) Nucleotide biases in all 23 to 30-nt sequences derived from YFV in *Dcr-2* null sibling mosquitoes*.*

We have previously published data showing that YFV replication increases in a *Dcr-2* null background (46). While this result is generally supportive of an antiviral role for the siRNA pathway in *A. aegypti*, the previous work did not address the importance of RNAi pathways in controlling the pathogenicity of the infection. Similar to results obtained with SINV, *Dcr-2* null *A. aegypti* infected with YFV exhibited significant mortality in comparison to wild-type siblings infected with equivalent virus titers (Fig. 4*A*). Further, *Dcr-2* null mutants infected with DENV-2 or DENV-4 also display a disease phenotype, exhibiting significantly elevated levels of virus replication and mortality in comparison to cohorts of wild-type siblings challenged with the same pathogens (*SI Appendix*, Fig. S7). While the biogenic products of the antiviral siRNA pathway are clearly visible in wild-type siblings infected with YFV, evidence of processing by the piRNA pathway is much less obvious (Fig. 4*B*). Notably, YFV-derived small RNAs that are 23 to 30 nucleotides in length, the size range most frequently associated with piRNAs, do not accumulate to the levels previously observed in adult mosquitoes and cell lines that were infected with alphaviruses (Figs. 2*C* and 4*B*) (19–22). However, a larger population of small RNAs mapping to the YFV genome, 30 to 40 nucleotides in length, are present in the wild-type sibling mosquitoes. These small RNAs exhibit a peak centered around 35 nt (Fig. 4*B*), rather than the 27 to 29 nucleotide peak previously observed in mosquitoes infected with SINV (Fig. 2*C*). Similar to vpiRNAs, the larger 30 to 40 nucleotide products are primarily derived from the positive-strands of the virus (Fig. 4*B*). Interestingly, biogenesis of vpiRNAs (47) was much more evident in *Dcr-2* null mutants infected with YFV, as indicated by the appearance of 23 to 30-nt products derived from the virus that exhibit a clear peak centered around 27 to 29 nucleotides in length. However, virus-derived small RNAs > 30 nt also accumulated in greater abundance in these mosquitoes (Fig. 4*C*).

Thus, we analyzed the YFV-derived small RNAs that were 23 to 30 nucleotides in length for any specific nucleotide biases or signatures of overlapping sequences. While we failed to detect any evidence for the production of vpiRNAs in the wild-type sibling mosquitoes infected with YFV (Fig. 4 *D* and *E*), small RNAs derived from YFV in the *Dcr-2* null mosquitoes exhibited clear hallmarks of ping-pong biogenesis (Fig. 4 *D* and *F*). However, similar to results obtained with SINV, the increased accumulation of vpiRNAs in the *Dcr-2* null mosquitoes was not sufficient to prevent increases in mortality resulting from virus infection (Fig. 4*A*). In summary, these results demonstrate that the production of siRNAs mediate an antiviral response in *A. aegypti*, which effectively modulates the pathogenicity of multiple human flavivirus pathogens in this host.

## Discussion

The natural maintenance cycles of arboviruses are characterized by the establishment of persistent, nonpathogenic infections in the invertebrate host, which for many viruses of public health importance is a mosquito (48). Mosquito-borne viruses that negatively affected the evolutionary fitness of the invertebrate vector host would presumably be disruptive to the complex transmission cycles of these pathogens, and unlikely to persist in nature. We show here that Dcr-2 is an essential host factor mediating an antiviral response in *A. aegypti* that prevents important pathogenic viruses from causing disease in the vector host. We found that *A. aegypti* defective for the production of siRNAs exhibit high viral loads and rapidly succumb to infection with multiple pathogens in the *alphavirus* and *flavivirus* genera (Movie S1 and *SI Appendix*, Fig. S7 and Figs. 2–4). These results demonstrate that the pathogenic effects of arboviruses in *A. aegypti* are primarily controlled by a canonical siRNA pathway, which functions as a resistance mechanism.

The concept of tolerance originally gained prominence in the field of plant ecology as the second component of host defense, the other being resistance, that determines the health of the plant during interactions with parasites (49–54). More recently, the model has been applied to study the defenses of animals to pathogens (55–57). In its simplest form, the model is based upon the very reasonable assumption that at some point increases in the intensity of infection will be inversely correlated with the health of the host (56, 57). The size of this decrease in health, or the fitness of the organism, represent the virulence associated with the infection (57). In principle, the virulence of the infection may be determined by factors associated with either the parasite or host, or more likely a combination of both (57). These factors may influence either the intensity of the infection or the extent of the damage caused by an individual parasite. Host factors that are involved in limiting the growth of the pathogen are said to contribute to the resistance of the organism to that particular pathogen, while host factors involved in surviving the infection, at a particular pathogen load, are said to contribute to the tolerance of that organism for that pathogen (55, 57). Note that both resistance and tolerance mechanisms are capable of modulating the negative effects of an infection on the health and fitness of an organism, but this is achieved through fundamentally different processes. A key distinction between the two mechanisms is that resistance limits pathogen replication, and thereby the parasite burden, whereas tolerance does neither. Rather, organisms exhibiting higher tolerance for a pathogen will exhibit smaller decreases in health as parasite burden increases (55, 57). Given the long-observed ability of disease vector mosquitoes to limit the health effects of infection with arboviruses, presumably without preventing infection or controlling virus replication, the idea that tolerance plays an important role in limiting fitness effects during infection in this particular host–parasite relationship is seductive.

To be clear, our results should not be interpreted as indicating that tolerance mechanisms do not play any role in determining the pathological outcome of arbovirus infections. In fact, quite the contrary, our data indicate that some *Dcr-2* null *A. aegypti* survived infection longer than others, despite being infected with identical virus genotypes at equivalent titers (Movie S1 and *SI Appendix*, Fig. S7 and Figs. 2*B*, 3*D*, and 4*A*). Indeed, additional parsing of the data shown in Fig. 3*C* indicated that viral loads in longer lived *Dcr-2* null mutants did not significantly differ from those in mutants that succumbed earlier (*SI Appendix*, Fig. S6). Thus, in the absence of a functional antiviral RNAi pathway, genetic variation in tolerance traits might explain the observed differences in longevity. However, according to the theory currently supported in plants, resistance and tolerance should be under correlational selection as they are mutually redundant traits (58). For example, an individual with high levels of resistance to a pathogen should have low levels of tolerance to the same pathogen, and vice versa. This is because individuals that are already highly resistant to a pathogen will benefit very little (e.g., very modest gains in fitness) if at all from increased tolerance, with the reverse also being true (i.e., a highly tolerant individual has little to gain from increases in resistance) (58). Our results show that *A. aegypti* modulates the pathogenesis of medically important viruses during systemic infections primarily through a Dcr-2-mediated resistance mechanism that limits virus replication. However, more modest gains in fitness may result from tolerance mechanisms present in this disease vector. Similarly, the production of vpiRNAs also may contribute to the fitness of the insect host in a secondary role.

In contrast with tolerance, the control of infections through resistance, is generally associated with the evolution of antagonistic mechanisms in pathogens. By directly limiting pathogen numbers, resistance mechanisms select for pathogens that evolve countermeasures capable of overcoming, evading, or suppressing the resistance mechanism (55). This suggests that viruses having evolved antagonistic mechanisms capable of overcoming the resistance-mediated immune defenses of *A. aegypti* should have been selected for in nature. Consistent with this, multiple arboviral sequences or proteins have been postulated to function as suppressors of the RNA silencing response (16). Conversely, the presence of such antagonistic mechanisms in pathogens would be predicted to exert a selective pressure on the host, driving the coevolution of more effective resistance mechanisms (55), which is consistent with studies demonstrating that *Dcr-2* is rapidly evolving in *A. aegypti* (59–61). In contrast, because tolerance mechanisms do not act by limiting infection intensity or pathogen burden, there is little selection pressure on pathogens to coevolve antagonistic methods to subvert tolerance (55). Rather, as tolerance traits mitigate the pathogen’s effects on the health and fitness of the host, without limiting the growth of the parasite, there should be either no selection (neutral) or possibly positive selection on the pathogen. In either case, this would result in a tendency for tolerance traits, unlike those involved in resistance, to become fixed in a host population over time (55).

This study contributes to evidence that RNAi pathways impact the health of humans, animals, and plants, by exerting an effect on the transmission of viral diseases. The roles that Dicer proteins play in controlling or limiting the pathogenicity of both animal and plant viruses, many of which are also transmitted by insect vectors, is also remarkably similar (47). In the future, the role of these pathways in the vertical transmission of arbovirus pathogens should also be investigated. Vertical transmission, involving parent-to-offspring passage of the virus, is more common among certain groups of arboviruses, suggesting that it may be a virus-specific phenomenon (62). For example, members of the *Flaviviridae* and *Peribunyaviridae* often exhibit this type of transmission, while it appears less common, or nonexistent, among members of the *Alphavirus* genus (62). However, this mechanism of transmission, in which mosquitoes serve as a reservoir, may also be an important factor in the maintenance of some arboviruses in nature (62). Antiviral immune pathways might have some role in regulating this type of transmission, but the possibility remains largely unexplored.

## Supplementary Material

Appendix 01 (PDF)

Movie S1. **Pathogenic alphavirus infection in RNAi deficient *Ae. aegypti.*** Side by side time course comparison of *Dcr-2* null mutant and wild-type sibling mosquitoes after infection with μ500 pfu of Sindbis virus.

## Data Availability

All of the small RNA datasets that were used in this study are available for download through the National Center for Biotechnology Information/Sequence Read Archive, accession number PRJNA691676 (63). All study data are included in the article and/or *SI Appendix*.
