# Supplementary material for: RNA interference is essential to modulating the pathogenesis of mosquito-borne viruses in the yellow fever mosquito Aedes aegypti
Source: Proc Natl Acad Sci U S A. 2023 Mar 9;120(11):e2213701120. doi: 10.1073/pnas.2213701120 (PMC10089172; doi:10.1073/pnas.2213701120)
Supplement: Supplementary file 1 — Appendix 01 (PDF) [file pnas.2213701120.sapp.pdf]

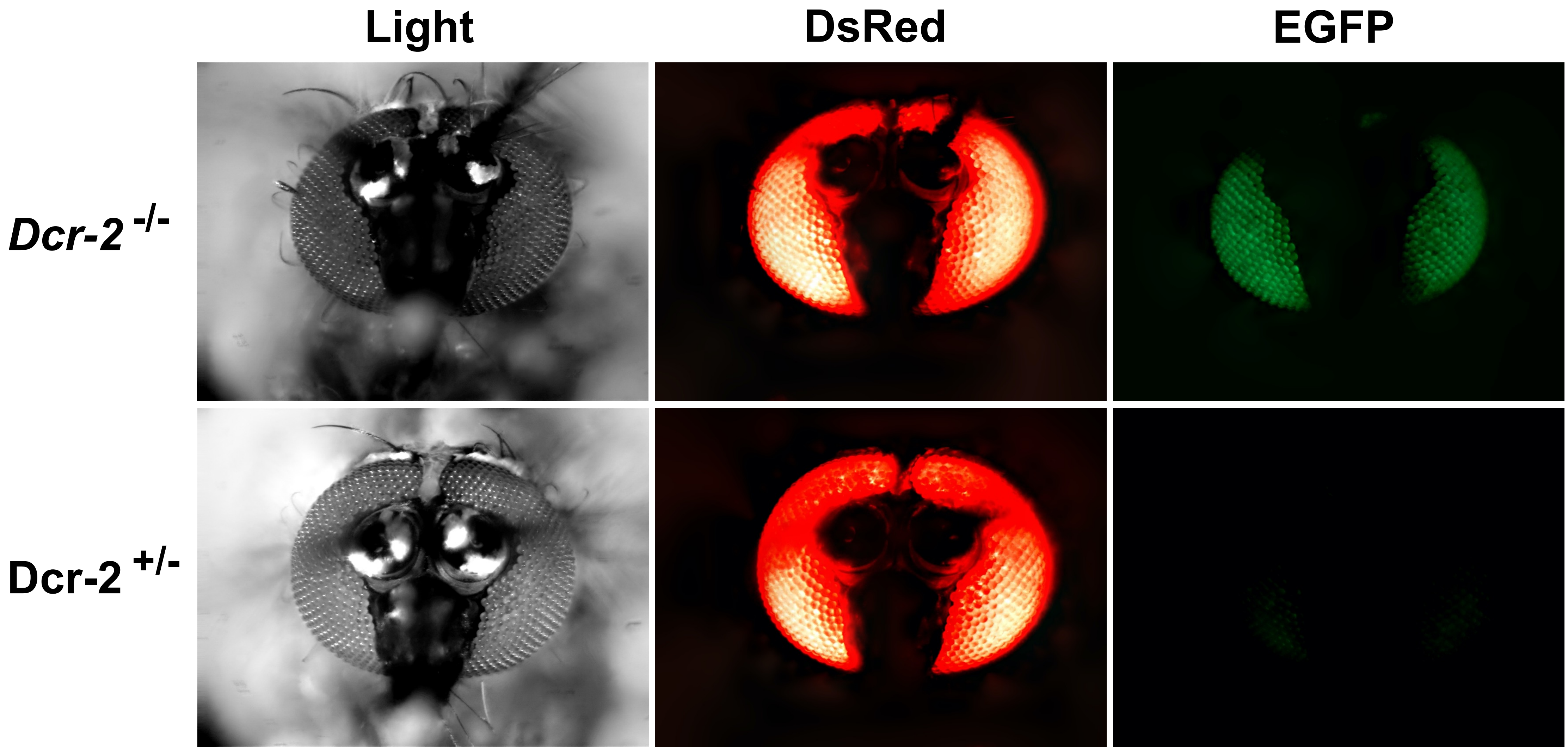

Fig. S1. The *Ae. aegypti Dcr-2* gene is haplosufficient. Representative images of sibling transgenic "sensor" mosquitoes that are either homozygous **(top row)** or heterozygous **(bottom row)** for mutant *Dcr-2* alleles. The images show expression of DsRed indicating that both sibling *Ae. aegypti* contain the transgenic "sensor" construct. Mosquitoes homozygous for the mutant alleles **(top row)** are unable to efficiently process the GFP-specific dsRNA substrate formed by the inverted repeat sequence expressed from the "sensor" construct, and effective silencing of GFP is prevented, resulting in the EGFP+ phenotype shown. However, mosquitoes with heterozygous *Dcr-2* alleles (i.e., one wild-type and one mutant allele; **bottom row**) effectively silence GFP expression in the transgenic "sensor" line, indicating that only a single normal allele provides enough functional protein to effectively silence EGFP expression from the "sensor" construct, resulting in the dark eyed phenotype shown.

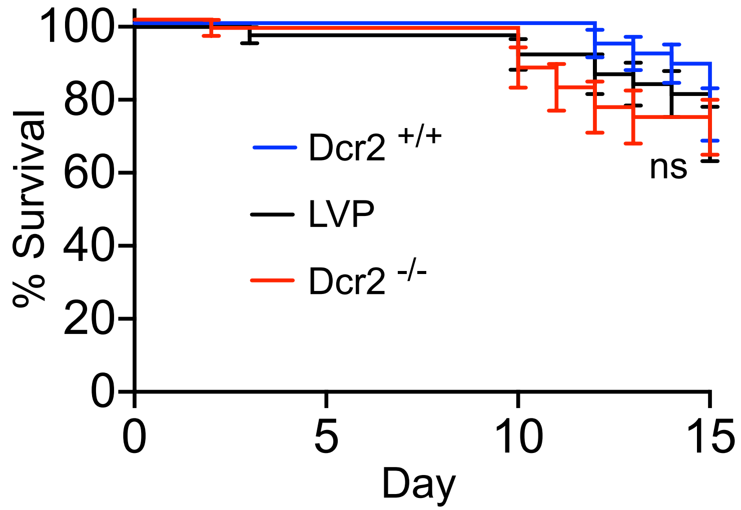

Fig. S2. The *Dcr-2* loss of function mutation has little or no adverse effect on the vigor of *Ae. aegypti* in the absence of microbial challenge. Survival of *Dcr-2* null (red line) or wild-type sibling (blue line) mosquitoes. The survival of Liverpool strain mosquitoes is shown for comparison (black line). Survival curves represent cohorts of ≥ 30 adult female mosquitoes, which were screened 7-10 days after emergence. The survival of homozygous *Dcr-2* null mutants did not significantly differ from those of wild-type siblings or Liverpool strain mosquitoes, as determined by a Mantel-Cox log-rank test.

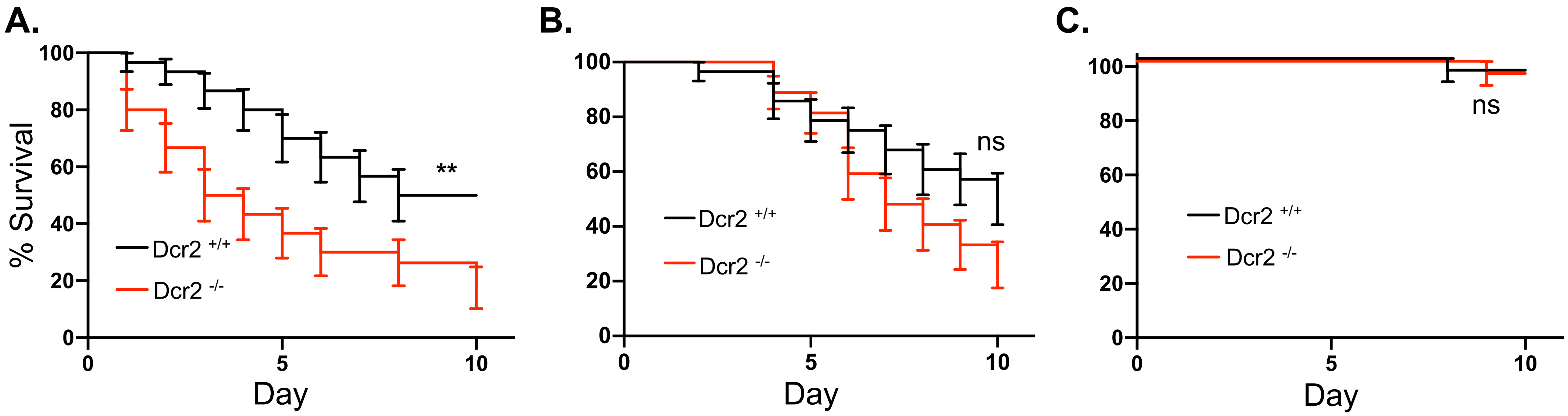

Fig. S3. *Dcr-2* null mutants demonstrate increased susceptibility to the Gram-positive bacteria, *S. aureus.* (A) Survival of *Dcr-2* null (red line) or wild-type sibling (black line) mosquitoes infected with S. aureus. **(B)** Survival of *Dcr-2* null (red line) or wild-type sibling (black line) mosquitoes infected with Gram-negative E. coli. **(C)** Survival of *Dcr-2* null (red line) or wild-type sibling (black line) mosquitoes mock infected with 1X PBS. Survival curves represent cohorts of 30 adult female mosquitoes injected with OD600 = 2.0 of bacteria. Significance was determined by a Mantel-Cox log-rank test, with (ns) representing not significant and (**) indicating a p value < 0.01.

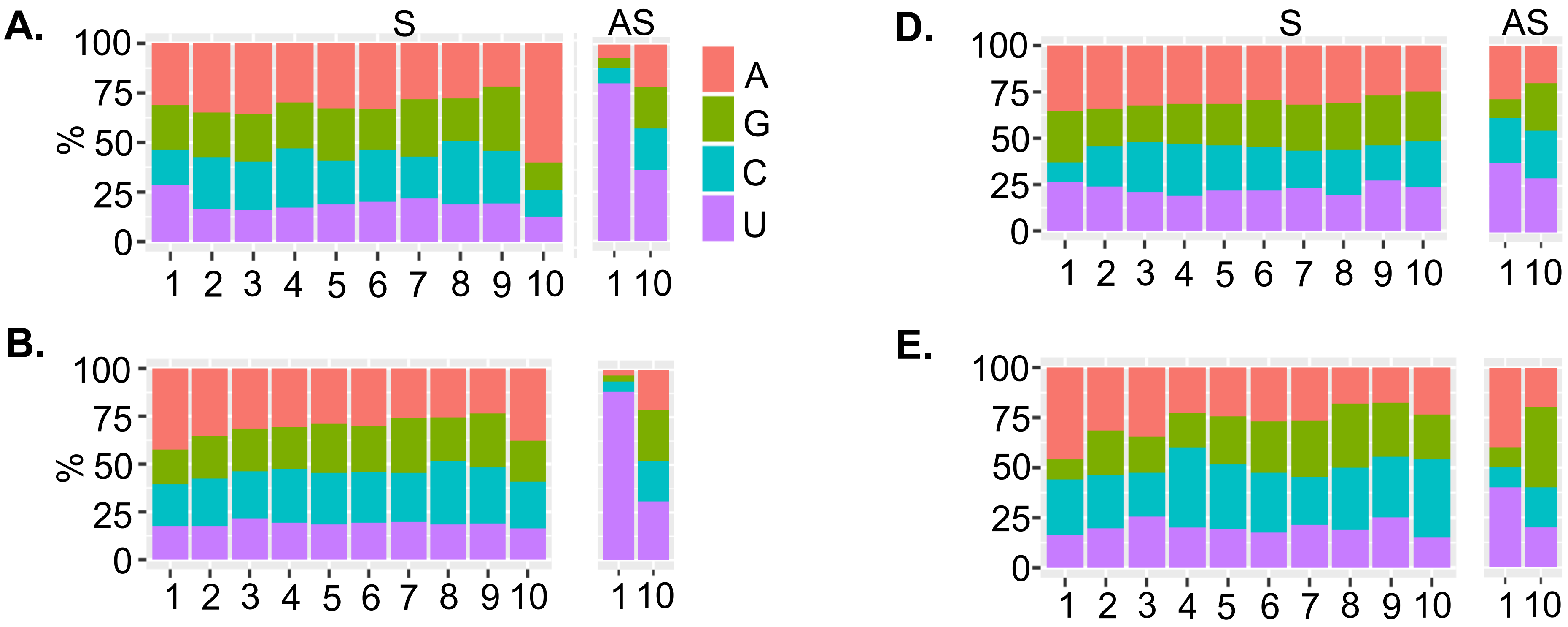

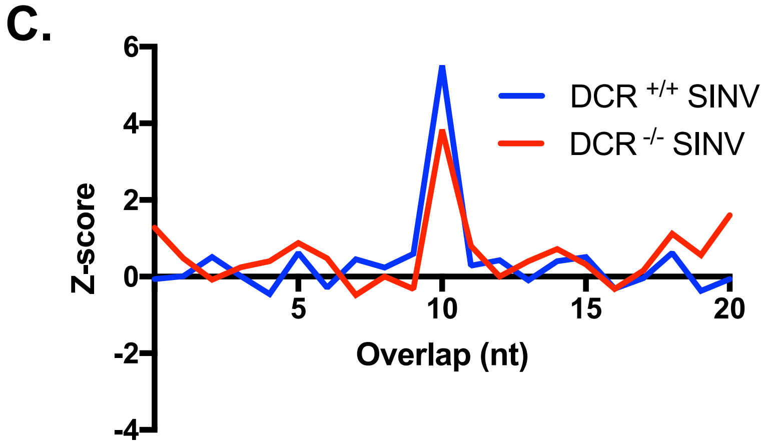

Fig. S4. Nucleotide biases and overlapping small RNA pairs in reads mapping to SINV. (A) Stacked bar graph showing individual nucleotide biases present at the positions indicated in all 23 to 30-nt sequences derived from sense (S) or anti-sense (AS) strands of SINV in wild-type sibling mosquitoes. **(B)** Nucleotide biases in all 23 to 30-nt sequences derived from SINV in *Dcr-2* null mosquitoes. **(C)** Z-scores for the number of small RNA pairs with the length of overlap indicated, determined from all 23 to 30-nt reads mapping to SINV in either wild-type (shown in blue) or *Dcr-2* null (shown in red) sibling mosquitoes. **(D)** Nucleotide biases in all 21-nt sequences derived from SINV in wild-type sibling mosquitoes. **(E)** Nucleotide biases in all 21-nt sequences derived from SINV in *Dcr-2* null mosquitoes.

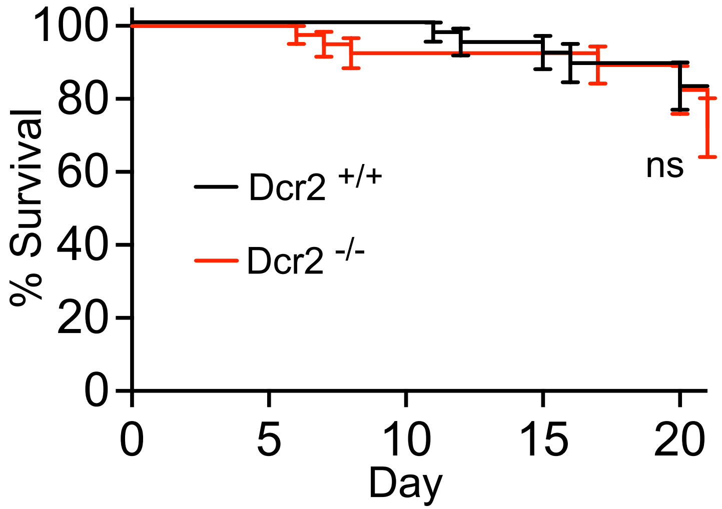

Fig. S5. Survival of homozygous *Dcr-2* null mutant *Ae. aegypti* after censoring those with systemic infections. Survival of *Dcr-2* null (red line) or wild-type sibling (black line) mosquitoes infected with SINV. Survival curves represent cohorts of ≥ 30 adult female mosquitoes following *per os* challenge with 6.7 x 10^6 pfu/ml of SINV. Significance was determined by a Mantel-Cox log-rank test, with (ns) representing not significant.

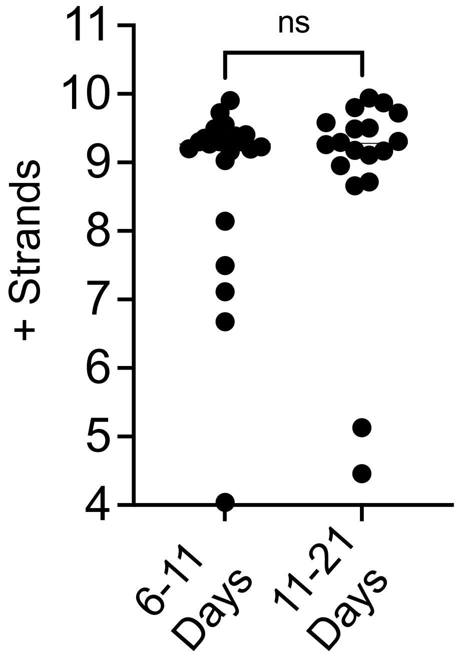

Fig. S6. RT-PCR results quantifying positive strand viral RNA (+ strands) or viral load in *Dcr-2* null mosquitoes with systemic SINV infections. Scatter plots of base values obtained from individual mosquito bodies (n ≥ 18). The X-axis indicates the time frame in which individual mosquitoes succumbed to the viral infection. The Y-axis is labelled with log10 values. Significance was determined by two-tailed t test performed on base values, with (ns) indicating not significant.

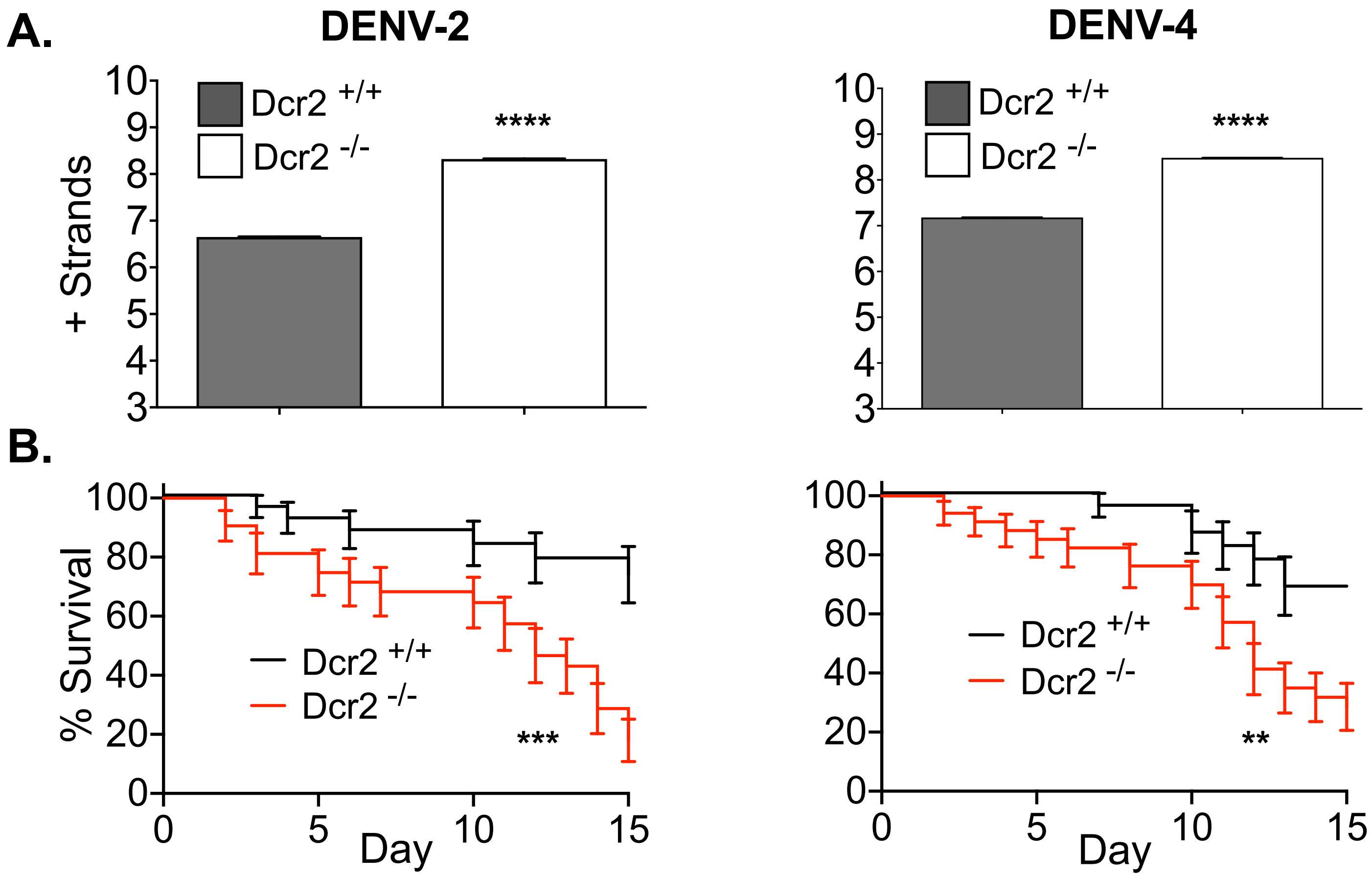

Fig. S7. The siRNA pathway modulates the pathogenesis of DENV-2 and DENV-4 infections in *Ae. aegypti*. (A) RT-PCR results showing the accumulation of positive strand viral RNA (+ strands), an indication of viral load, in wild-type or *Dcr-2* null sibling mosquitoes 4 days after infection with either DENV-2 or DENV-4 (~500 pfu injected into individual mosquitoes). The Y-axis is labelled with log10 values. Error bars indicate the SEM calculated from three independent biological replicates (n = 5). Significance was determined by two-tailed t test performed on base values, with (****) indicating a p value < 0.0001**. (B)** Survival of wild-type (black line) or *Dcr-2* null (red line) sibling mosquitoes infected with either DENV-2 or DENV-4. Survival curves represent cohorts of ≥ 30 adult female mosquitoes injected with ~500 pfu of virus. Significance was determined by a Mantel-Cox log-rank test, with (***) indicating a p value < 0.001 and (**) indicating a p value < 0.01.

Movie S1. Pathogenic alphavirus infection in RNAi deficient *Ae. aegypti*. Side by side time course comparison of *Dcr-2* null mutant and wild-type sibling mosquitoes after infection with ~500 pfu of Sindbis virus.
